# Chronological analysis of the gut microbiome for efficacy of atezolizumab‐based immunotherapy in non‐small cell lung cancer: Protocol for a multicenter prospective observational study

**DOI:** 10.1111/1759-7714.14640

**Published:** 2022-09-05

**Authors:** Fumihiro Shoji, Naoko Miura, Tetsuzo Tagawa, Shuichi Tsukamoto, Tatsuro Okamoto, Koji Yamazaki, Motoharu Hamatake, Sadanori Takeo

**Affiliations:** ^1^ Department of Thoracic Surgery Clinical Research Institute, National Hospital Organization, Kyushu Medical Center Fukuoka Japan; ^2^ Department of Thoracic Oncology National Hospital Organization, Kyushu Cancer Center Fukuoka Japan; ^3^ Department of Thoracic Surgery Saiseikai Fukuoka General Hospital Fukuoka Japan; ^4^ Department of Surgery and Science Kyushu University Fukuoka Japan; ^5^ Department of Thoracic Surgery Kitakyushu Medical Center Kitakyushu Japan; ^6^ Department of Thoracic Surgery Steel Memorial Yawata Hospital Kitakyushu Japan

**Keywords:** atezolizumab‐based immunotherapy, gut microbiome, non‐small cell lung cancer, prospective observational study

## Abstract

**Background:**

Cancer immunotherapy with immune checkpoint inhibitors (ICIs) is an innovative treatment for non‐small cell lung cancer (NSCLC). Recently, the specific composition of the gut microbiome before initiation of cancer immunotherapy has been highlighted as a predictive biomarker in patients undergoing cancer immunotherapy, mainly in the US or Europe. However, the fact gut microbiome status is completely different in races or countries has been revealed. In addition, how the microbiome composition and diversity chronologically change during cancer immunotherapy is still unclear.

**Methods:**

This multicenter, prospective observational study will analyze the association between the gut microbiome and therapeutic response in NSCLC patients who received atezolizumab‐based immunotherapy. The aim of the present study is to clarify not only how the specific composition of the gut microbiome influences clinical response in NSCLC patients but the chronological changes of gut microbiota during atezolizumab‐based immunotherapy. The gut microbiota will be analyzed using 16S rRNA gene sequencing. The main inclusion criteria are as follows: (1) Pathologically‐ or cytologically‐confirmed stage IV or postoperative recurrent NSCLC. (2) Patients ≥20 years old at the time of informed consent. (3) Planned to treat with atezolizumab‐based immunotherapy combined with platinum‐based chemotherapy (cohort 1) and monotherapy (cohort 2) as a first immunotherapy. (4) Patients to provide fecal samples. A total of 60 patients will be enrolled prospectively. Enrollment will begin in 2020 and the final analyses will be completed by 2024.

**Discussion:**

This trial will provide more evidence of how gut microbiota composition and diversity chronologically change during cancer immunotherapy and contribute to the development of biomarkers to predict ICI response as well as biotic therapies which enhance the ICI response.

## Graphical abstract

This multicenter, prospective observational study will analyze the association between the gut microbiome and therapeutic response in non‐small cell lung cancer (NSCLC) patients who received atezolizumab‐based immunotherapy. The aim of the present study is to clarify not only how the specific composition of the gut microbiome influences clinical response in NSCLC patients but the chronological changes of gut microbiota during atezolizumab‐based immunotherapy. A total of 60 patients will be enrolled prospectively and this trial will provide more evidence of how gut microbiota composition and diversity chronologically change during cancer immunotherapy and contribute to the development of biomarkers to predict immune checkpoint inhibitors (ICI) response as well as antibiotic therapies to enhance the ICI response.

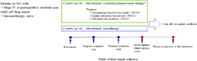

## INTRODUCTION

Cancer immunotherapy such as immune checkpoint inhibitors (ICI) targeting programmed cell death‐1 (PD‐1), programmed cell death‐ligand 1 (PD‐L1) and cytotoxic T lymphocyte‐associated protein‐4, is widely used to treat various malignancies, including non‐small cell lung cancer (NSCLC); it has changed therapeutic approaches to malignancies. PD‐L1 is an immune checkpoint protein expressed on both tumor and tumor‐infiltrating immune cells, which can mediate anticancer immunosuppression.^1^ Anti‐PD‐1 antibodies (e.g., nivolumab and pembrolizumab) and anti‐PD‐L1 antibodies (e.g., atezolizumab and durvalumab) enable T cell activation and immune system recognition.

Although tumorous PD‐L1 expression is well known as a conventional biomarker of the ICI therapeutic response, there is no widely accepted optimal biomarker to predict the efficacy of ICI, because ICI response and survival outcomes show heterogeneity in NSCLC patients receiving cancer immunotherapy, regardless of PD‐L1 expression level.

We recently reported that the pretreatment host immune‐nutritional condition was a prognostic biomarker for NSCLC patients treated with cancer immunotherapy.^2^ Host immunity is clearly associated with the ICI response and the internal microbiome is regarded as a controlling factor in host immunity. In particular, the gut microbiome can modulate the host immune response (e.g., antitumor immunity) and optimize both innate and adaptive immune responses.^3^ Recently, preclinical analyses have shown that the gut microbiota composition and its modification in murine models could influence the efficacy of ICIs.^4^, ^5^ Therefore, the microbiome has been emphasized as a predictive biomarker of cancer immunotherapy, mainly in reports from the US or Europe. In addition, gut microbiome diversity or abundance of specific gut microbiome components has been reported to be related to the efficacy of anti‐PD‐1 antibody in melanoma patients.^6^ Moreover, fecal microbiota transplantation (FMT) in murine models might restore the ICI response.^7^, ^8^ In a recent study, FMT from ICI responders to ICI nonresponders produced ICI efficacy in melanoma patients.^9^

FMT or antibiotic therapy approaches are needed to investigate changes in the microbiome during cancer immunotherapy. Notably, there are definite differences in microbiome composition among ethnicities.^10^ We recently reported that high gut microbiome diversity and specific composition such as the genus *Blautia* and order RF32 unclassified were significantly correlated with therapeutic response to cancer immunotherapy in Japanese NSCLC patients treated with cancer immunotherapy. However, this previous study highlighted only the gut microbiome during cancer immunotherapy including various ICIs. Therefore, how gut microbiota recognized before the initiation of cancer immunotherapy chronologically change during cancer immunotherapy is currently unknown. Here, we will perform a prospective observational study to clarify the time course of microbiota composition and diversity in Japanese NSCLC patients treated with cancer immunotherapy using a limited ICI (atezolizumab‐based immunotherapy) by analyzing gut microbiota collected chronologically.

## METHODS

### Study objectives

Our objective is to evaluate the association between the therapeutic response of atezolizumab‐based immunotherapy and gut microbiome in NSCLC patients and to clarify the relationship of gut microbiome and clinicopathological factors, outcome and immune‐related adverse events by analyzing gut microbiota chronologically during atezolizumab‐based immunotherapy.

The study has been approved by the institutional review boards at each participating center.

### Study setting

This is a multicenter, prospective observational study.

### Observational points

In the present study, we will clarify the following:

1. Six‐month progression‐free survival (PFS) according to the composition and diversity of gut microbiota
2. Twelve‐month overall survival (OS) according to the composition and diversity of gut microbiota
3. The incident rate of immune‐related adverse events (irAE) (≥grade 3) according to the composition and diversity of gut microbiota
4. Six‐month PFS according to the use of antibiotics
5. The association between the diversity and composition of gut microbiota, and the clinicopathological features including PD‐L1 expression, hematological data, immune‐nutritional indices, the use of antibiotics within 28 days before initiation of atezolizumab‐based immunotherapy, and the total number of atezolizumab‐based immunotherapy administered
6. Six‐month PFS of all patients
7. Twelve‐month OS of all patients
8. The response rate of atezolizumab‐based immunotherapy
9. Duration of response of atezolizumab‐based immunotherapy
10. Disease control rate of atezolizumab‐based immunotherapy
11. The incident rate of all grade of AEs
12. The analysis of efficacy and safely according to the following: Brain metastasis at baseline; interstitial pneumonia at baseline; the use of corticosteroids at baseline; sex; age at baseline; European Clinical Oncology group (ECOG) performance status at baseline; regimens of platinum‐doublet chemotherapy; the number of treatments and regimens after failure of atezolizumab‐based immunotherapy; the cycle number of atezolizumab‐based immunotherapy performed; the best response of atezolizumab‐based immunotherapy; tumor proportional score (tumoral PD‐L1 expression); previous history of autoimmune diseases; smoking history and driver mutation status.

### Data collection

**FIGURE 1:**
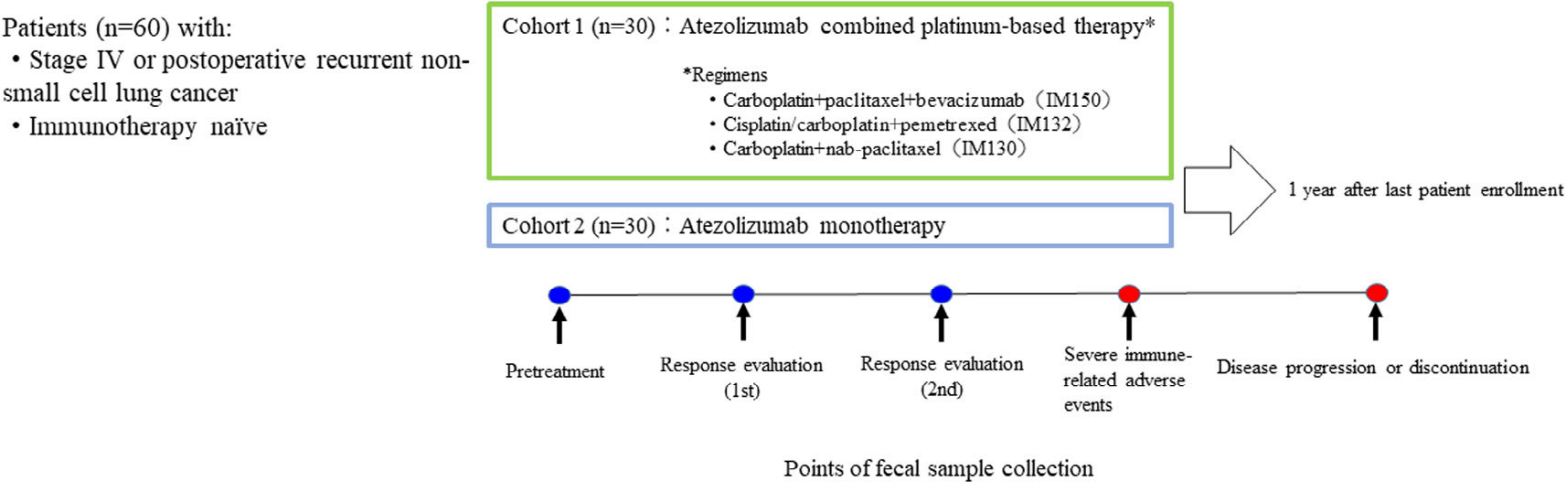
Study design

This study will consist of two cohorts (cohort 1 and cohort 2). Cohort 1 will include patients treated with atezolizumab combined platinum‐based therapy such as IMpower 150, IMpower 132 or IMpower 130 regimens.^11^, ^12^, ^13^ Cohort 2 will include patients treated with atezolizumab monotherapy.^14^ Patients are followed over time including baseline (pretreatment period), first response evaluation (period within 24 weeks after treatment initiation), second response evaluation (period before/after administration of eighth cycle treatment), periods at the occurrence of severe immune‐related adverse events (irAEs) and periods at progression of disease or discontinuation of this treatment. Stool and blood samples are collected at each period to correlate the clinical/graphical response to atezolizumab‐based immunotherapy, severity or contents of irAE, and continuation/discontinuation of treatment with an individual's gut microbiome. The present study design is shown in Figure 1.

### Eligibility criteria

Inclusion criteria: (i) Pathologically‐ or cytologically‐confirmed stage IV or postoperative recurrent NSCLC. (ii) Patients ≥20 years old at the time of informed consent. (iii) Planned to treat with atezolizumab‐based immunotherapy combined with platinum‐based chemotherapy (cohort 1) and atezolizumab‐monotherapy (cohort 2) as a first immunotherapy. (iv) Patients to provide fecal samples. (v) Patients to provide their written informed consent.

Exclusion criteria: (i) Patients deemed inappropriate for the study by the investigator.

### Sample size

The primary analysis of this study is to estimate the 6‐month PFS and 90% confidence interval (CI) based on normal approximation. In cohort 1, 26 patients were planned to be enrolled into this study, based on considering sufficient to estimate 90% confidence intervals for the true 6‐month PFS within a width of ± 0.15, when the true 6‐month PFS is expected to be 65%. In cohort 2, 27 patients were planned to be enrolled into this study, based on considering sufficient to estimate 90% confidence intervals for the true 6‐month PFS within a width of ± 0.15, when the true 6‐month PFS is expected to be 30%. Taking into account ineligible participants and those lost to follow‐up, the target sample size was determined to be 60 participants (30 participants for each cohort).

### Registration

The accrual started in October 2020.

### Study population

We are planning to recruit eligible participants from participant hospitals. All enrolled patients should have at least one measurable target lesion based on the Response Evaluation Criteria in Solid Tumors (RECIST), version 1.1.^15^ Clinical/pathological stage is based on the Tumor Node Metastasis (TNM) classification established by the International Union Against Cancer.^16^ For TNM staging, all patients undergo computed tomography (CT) of the thorax and upper abdomen, as well as bone scintigrams, brain CT scans, magnetic resonance imaging (MRI), or fluorodeoxyglucose‐positron emission tomography (FDG‐PET). Postoperative local or distant recurrence is defined as previously described.^17^ Atezolizumab‐based immunotherapy is continued until radiographic progression or discontinuation due to severe irAEs or patient request. PD‐L1 protein expression is evaluated using antibody clone 22C3 (Dako, Agilent Technologies). Adverse events are graded according to Common Terminology Criteria for Adverse Events (CTCAE) version 5.0.

### Treatment plan

Cohort 1 (*n* = 30): IMpower150 regimen: atezolizumab (1200 mg intravenously every 3 weeks) plus carboplatin (area under the concentration [AUC]–time curve of 6 mg/ml/min for 4 cycles) plus paclitaxel (200 mg/m^2^ intravenously every 3 weeks for 4 cycles) plus bevacizumab (15 mg/kg) intravenously every 3 weeks.^11^ IMpower130 regimen: atezolizumab (1200 mg intravenously every 3 weeks) plus carboplatin (AUC of 6 mg/ml/min every 3 weeks) plus nab‐paclitaxel (100 mg/m^2^ every week).^12^ IMpower132 regimen: atezolizumab (1200 mg intravenously every 3 weeks) plus cisplatin (75 mg/m^2^) or carboplatin (AUC of 6 mg/ml/min) plus pemetrexed 500 mg/m^2^ administered every 3 weeks.^13^ Maintenance therapy with atezolizumab plus bevacizumab (IMpower150), atezolizumab plus pemetrexed (IMpower132) or atezolizumab alone (IMpower130) given every 3 weeks after induction therapy until disease progression, unacceptable toxicity, or death. Cohort 2 (*n* = 30): Atezolizumab administered at a dose of 1200 mg intravenously every 3 weeks.^14^

### Sample collection, DNA extraction, gene amplification, sequencing, and data analysis procedures

Fecal samples are collected in sterile containers and immediately placed at 4°C, then frozen at −80°C. The preliminary treatment of fecal samples is conducted in accordance with a previously described method.^18^ DNA is then extracted using an automated DNA isolation system (Gene Prep Star PI‐480, Kurabo) from saliva using the Mora‐Extract kit (Kyokuto Pharmaceutical). The V3–V4 regions of bacterial 16S rRNA genes are amplified using the Pro341F/Pro805R primers^18^ and dual‐index method^19^ under hemi‐nested PCR conditions.^20^ Barcoded amplicons are paired‐end sequenced on a 2 × 284‐bp cycle using the MiSeq system with MiSeq reagent kit chemistry, version 3 (600 cycle), and paired‐end sequencing reads merged using the fastq‐join program with default settings.^21^

The joined amplicon sequence reads are processed through QIIME 2 version 2020.6.^22^ The quality value score of <33 and chimeric sequence are filtered and representative sequences created using DADA2 denoise‐single plugin version 2017.6.0.^23^ Taxonomy of representative sequences is assigned using Greengenes database version 13.8^24^ by training a Naive Bayes classifier using q2‐feature‐classifier plugin. Alpha diversity indices (chao1, Shannon and Simpson) are calculated alpha‐rarefaction plugin. The statistical significance of chao1, Shannon and Simpson indices among groups is assessed by Kruskal–Wallis test using alpha‐group‐significance plugin. Beta diversity is analyzed using weighted and unweighted unifrac, and Bray‐Curtis distances using the core‐metrics‐phylogenetic plugin. The Emperor tool is used to visualize principal coordinate analysis (PCoA) plots. The statistical significance of similarity of bacterial communities among groups is assessed by the analysis of similarities (ANOSIM) test using the beta‐group‐significance plugin. Heat map and Ward's clustering from phylum to species is shown using the feature‐table heatmap plugin.

### Statistical analysis

Categorical variables are analyzed using Fisher's exact test. Continuous variables are compared using the chi‐squared test. The Mann–Whitney U test is used to determine significant differences among the different groups using alpha diversity, which shows the diversity in each individual sample. Logistic regression analysis to calculate odds ratios for ICI response with respect to clinicopathological characteristics is used. Kaplan–Meier statistics and log‐rank testing to evaluate PFS is applied. Statistical analyses are performed using JMP software, version 14.0 (SAS Institute Inc.). *p‐*values <0.05 are considered statistically significant.

## DISCUSSION

This multicenter, prospective observational study will analyze the association between the gut microbiome and therapeutic response in NSCLC patients who received atezolizumab‐based immunotherapy. The aim of the present study is to clarify not only how the specific composition of the gut microbiome influences clinical response in Japanese NSCLC patients but the chronological changes of gut microbiota during atezolizumab‐based immunotherapy. Thus, we aim to contribute to not only provide more evidence of how gut microbiota composition and diversity chronologically change during cancer immunotherapy but the development of biomarkers to predict ICI response as well as antibiotic therapies to enhance the ICI response.

### Patient and public involvement

Patients and/or public were not involved in the design of the present study.

### Ethics and dissemination

Study results will be disseminated through peer‐reviewed journals and national and international conferences.

### Participating institutions

Department of Thoracic Surgery, Clinical Research Institute, National Hospital Organization, Kyushu Medical Center, Department of Thoracic Oncology, National Hospital Organization, Kyushu Cancer Center, Department of Thoracic Surgery, Saiseikai Fukuoka General Hospital, Department of Surgery and Science, Kyushu University, Department of Thoracic Surgery, Kitakyushu Medical Center, and Department of Thoracic Surgery, Steel Memorial Yawata Hospital.

## CONFLICT OF INTEREST

None declared.
